# Metformin Sustained-Release and Vildagliptin Fixed-Dose Combination for Optimizing Glycemic Control: A Review with Real-World Case Reports

**DOI:** 10.3390/clinpract13020045

**Published:** 2023-03-28

**Authors:** Manoj Chawla, Purvi Chawla, Pratap Jethwani, Kiran Shah, Sanjay Reddy

**Affiliations:** 1 Department of Endocrinology, SL Raheja Hospital, Mumbai 400016, Maharashtra, India; 2 Lina Diabetes Care, Diabetes & Beyond, Mumbai Diabetes Research Centre, Andheri, Mumbai 400053, Maharashtra, India; 3 PG Dip Diabetes, FRSSDI, F Diab India, Jethwani Hospital & Diabetes Care Center, Rajkot 360001, Gujarat, India; 4 Grant Medical College & Sir JJ Group of Hospitals, Diabetes and Thyroid Care Center, Mumbai 400092, Maharashtra, India; 5 Center for Diabetes and Endocrine Care Bangalore, Fortis Hospital Bangalore, Bangalore 560043, Karnataka, India

**Keywords:** type 2 diabetes mellitus, metformin, vildagliptin, fixed-dose combination, sustained release, compliance, tolerability, adherence, early combination therapy

## Abstract

(1) Background: There is a high burden of poor glycemic control in the Indian population with type 2 diabetes mellitus (T2DM). Currently, the use of metformin sustained-release (SR)–vildagliptin fixed-dose combination (FDC) is very low as compared to metformin immediate-release (IR)–vildagliptin FDC which is associated with higher adverse events (AEs). Here, we present real-world effectiveness of metformin SR–vildagliptin FDC treatment in patients with T2DM; (2) Methods: This retrospective analysis was carried out from the medical records of adult T2DM patients visiting a single study center in India (December 2020–February 2021). A total of 10 patients (aged ≥20 years) were treated with vildagliptin 50 mg and metformin SR 500 mg FDC for 15 days. The treatment response was assessed by the percentage of time spent in the target glucose range (TIR at baseline and 15 days after treatment); (3) Results: The glycated hemoglobin (HbA1c) levels at baseline varied between 6.5% to 12%. The glycemic control improved in 70% of patients (mean increase in TIR: 18.9%). Treatment adherence was 100%. No gastrointestinal symptoms or AEs were reported; (4) Conclusions: Early intervention with metformin SR–vildagliptin FDC in patients with T2DM can ensure therapy compliance in terms of superior efficacy along with safety and tolerability. Key summary points: Early initiation of combination therapy helps in early achievement of glycemic goals; Early initiation of metformin and vildagliptin FDC results in significant glycemic control with good tolerability and compliance; Metformin SR–vildagliptin FDC has lower adverse events, compared to metformin IR–vildagliptin FDC; A case series of ten patients with T2DM treated with metformin SR–vildagliptin FDC is presented to assess the real-world effectiveness of this combination.

## 1. Background

### 1.1. Burden of Diabetes and Its Complications

Similar to the global scenario, the burden of diabetes in India has steadily increased over the past few decades. India has been a major contributor to the global burden of diabetes [1]. Diabetes affected 74.2 million adults aged ≥20 years in India in 2021, and this figure is expected to rise to 124.9 million by 2045 [1]. The Investigation of Glycosylated Hemoglobin on Therapy in Indian diabetics (TIGHT) study revealed that despite receiving antidiabetic therapy, there is a high burden of poor glycemic control in the Indian population with type 2 diabetes mellitus (T2DM). As high as 76.6% of the T2DM population already on antidiabetic therapy was found to have glycated hemoglobin (HbA1c) levels ≥ 7.0%, and the mean HbA1c was 7.7%. One-third of this population had diabetes-associated microvascular complications. These findings emphasize the need for early implementation of appropriate pharmacotherapy in the Indian population with T2DM to achieve recommended glycemic targets, thereby lowering the concomitantly increasing burden of microvascular complications [2].

### 1.2. Current Unmet Needs in the Management of Glycemic Control

Conventionally, management of T2DM follows a stepwise intensification approach wherein lifestyle modification is followed by a single-agent oral antidiabetic drug (OAD) and combination therapy. Although this approach helps in achieving glycemic control, it has several limitations including delays in achieving glycemic targets and in switching from monotherapy to combination therapy [3]. In light of such limitations, early initiation of combination therapy helps in earlier achievement of glycemic goals, sustained glycemic control, better preserves β-cell function, and delays the deterioration of glycemic control [4,5]. The American Diabetes Association guidelines recommend early initiation of combination therapy to extend the time to treatment failure [6]. In India, the use of combination therapy is high (91%) among the population with diabetes, and the use of dual combination therapy is much higher than oral antihyperglycemic agents (76% vs. 15%) [7].

The formulation of multiple oral hypoglycemic agents in a single-dose form called fixed-dose combination (FDC) has an important role in glycemic control [8]. The advantages of FDC therapy in T2DM management include lowering pill burden and improving glycemic control with better efficacy and better treatment adherence [8]. Patients who are intolerant to metformin or who experience side effects of metformin monotherapy are given FDCs of various OADs such as dipeptidyl peptidase (DPP-4) inhibitors, sodium–glucose cotransporter-2 (SGLT-2) inhibitors, glucagon-like peptide-1 (GLP-1) antagonist, thiazolidinediones (TZDs), and sulfonylureas (SUs). For enhancing treatment efficacy, the addition of a third agent to FDC can also be considered [3].

Over the past decades, Hb_A1c_ was used as the primary metric for evaluating glucose management and the efficacy of diabetes care. Although Hb_A1c_ retrospectively captures the average glycemic control, this metric has some limitations, such as incapability to capture day-to-day glucose fluctuations and assess short-term outcomes. Moreover, Hb_A1c_ does not provide information about severe hyperglycemia or hypoglycemia. Self-monitoring of blood glucose (SMBG) has considerably improved glycemic control by aiding individual adjustment of insulin dosing. However, SMBG provides intermittent fragments of real glucose fluctuations, thereby failing to monitor ongoing glucose fluctuations even with frequent use. All these limitations associated with the use of Hb_A1c_ and SMBG are addressed by continuous glucose monitoring (CGM), which provides a broad spectrum of glucose management metrics including the proportion of time in range (TIR), time above range (TAR), time below range (TBR), and glycemic variability, thereby allowing individualized diabetes care and real-time treatment modifications [9]. For the majority of people with T2DM, a TIR (between 70 and 180 mg/dL) target of >70% is recommended, with every 5% increase toward this target being clinically meaningful.

## 2. Metformin SR–Vildagliptin FDC Therapy: Evidence-Based Rationale

### 2.1. Metformin IR–Vildagliptin FDC Therapy

Vildagliptin is a selective and reversible inhibitor of the DPP-4 enzyme that inactivates the incretin hormones, including GLP-1, and glucose-dependent insulinotropic polypeptide hormones, which significantly contribute to the maintenance of glucose homeostasis [10]. In a double-blind, randomized, multicenter, parallel-group, 24-week trial in patients with T2DM inadequately controlled on metformin therapy, vildagliptin improved glycemic control was well tolerated, and produced clinically meaningful, dose-related decreases in HbA1C and fasting plasma glucose as add-on therapy to metformin [11]. The metformin immediate-release (IR)–vildagliptin FDC confers superior efficacy by demonstrating an additive effect on plasma glucose lowering, along with a beneficial effect on β-cell function [12].

Until recently, metformin was available as an IR formulation that was required to be taken thrice a day, at dosages of 500 mg, 850 mg, or 1000 mg [13]. Besides multiple dosing, i.e., 2–3 times per day, metformin IR therapy is associated with numerous GI AEs [13,14,15], which leads to treatment nonadherence among patients [14,16,17] and raised HbA^1c^ levels [17], making dose optimization challenging for physicians [18]. The metformin IR–vildagliptin FDC elicits the common problems associated with metformin IR monotherapy, despite the FDC being associated with better glycemic control than metformin monotherapy [4,15].

### 2.2. Role of Metformin SR Therapy in Improved Treatment Satisfaction

Metformin sustained-release (SR) therapy was introduced to address all of the aforementioned limitations associated with metformin IR therapy [18,19].

**Figure 1:**
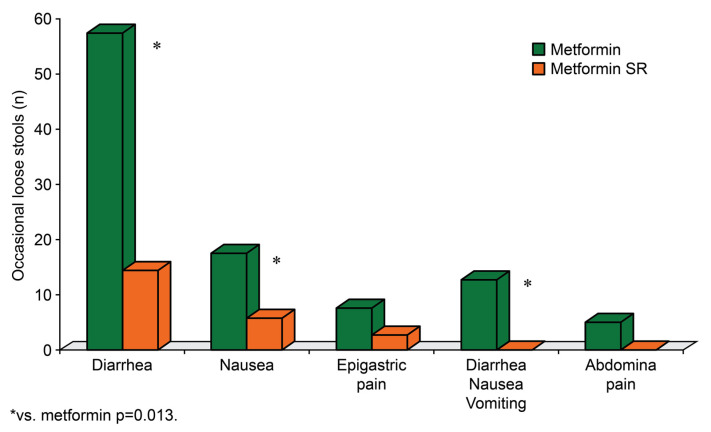
Gastrointestinal disturbance with metformin SR vs. metformin (immediate release); SR: Sustained release. Adapted from Levy et al., 2010 [21].

In contrast to metformin IR, which releases 90% of the drug within 30 min, metformin SR delays time to peak plasma concentrations by 4–7 h, has a longer gastric residence, and is absorbed more slowly from the upper GI tract. Thus, it demonstrates improved GI tolerability with convenient once-daily dosing [20]. After 6 months of continuous once-daily therapy, metformin SR demonstrated no GI AEs in 77% of patients, and 83% of patients preferred metformin SR at the end of the therapy period (Figure 1) [21].

Metformin SR therapy in Asian patients for 12 weeks demonstrated that only 3.3% of patients experienced ≥1 GI side effect, which implied that around 96.7% of patients were devoid of GI AEs with metformin SR therapy [20]. Switching to metformin SR from IR was associated with a >50% reduction in the incidence of GI events (26.34% to 11.71%, *p* = 0.0006) [22] and improved treatment adherence (>90% adherence) along with improved glycemic control [23]. Therefore, owing to the higher convenience of treatment, metformin SR is strongly preferred by patients over metformin IR [23].

Both metformin and vildagliptin have a complementary mechanism of action and possibly exert synergistic effects on glycemic control. Therefore, a bilayer tablet of this FDC was developed containing metformin SR layer and vildagliptin IR layer to lower GI side effects of metformin, reduce dosing frequency, and improve bioavailability and patient compliance [15].

## 3. Clinical and Real-World Benefits of the Metformin SR–Vildagliptin FDC

### 3.1. Efficacy, Safety, and Therapy Compliance with the FDC of Metformin and Vildagliptin: Clinical Evidence

The Vildagliptin Efficacy in combination with metfoRmIn For earlY treatment of type 2 diabetes (VERIFY) trial demonstrated a significant risk reduction for time to initial treatment failure in newly diagnosed patients with T2DM in the early metformin–vildagliptin combination therapy group vs. the metformin monotherapy group over the 5-year study duration (*p* < 0.0001) [24,25].

The Initial combiNation therapy with vildagliptin plus metformin In drug-naïve T2DM patients In a reAl Life setting (INITIAL) study demonstrated significant reductions in Hb_A1c_ both at weeks 12 and 24 (−1.6% and −1.9%, respectively; *p* < 0.001) after initial treatment with vildagliptin–metformin combination in Asian patients with T2DM who presented with high baseline HbA1c (9.3% ± 1.6%) and multiple CV risk factors [26].

### 3.2. Sustained-Release vs. Immediate-Release Metformin and Vildagliptin Combination

A recent study in the Indian setting reported that metformin IR–vildagliptin combination is associated with higher AEs such as gastric discomfort, acidity, nausea, and indigestion as compared to metformin SR–vildagliptin combination. Even for metformin monotherapy, metformin IR is associated with higher AEs than metformin SR in FDC therapy [4]. Further, there is a scarcity of data in the Indian context on the efficacy of metformin SR–vildagliptin FDC in T2DM management. In this context, we provide real-world case reports of T2DM patients treated with metformin SR–vildagliptin FDC to assess the effects on TIR.

## 4. Real-World Effectiveness of Metformin SR–Vildagliptin FDC: Case Presentation

### 4.1. Objective

To evaluate the real-world effectiveness of metformin SR–vildagliptin FDC in T2DM patients.

### 4.2. Methods

This retrospective analysis was carried out from the medical records of adult T2DM patients visiting a single study center in India during the period December 2020–February 2021. Only those patients with T2DM aged ≥20 years, treated with metformin SR–vildagliptin FDC (vildagliptin 50 mg and metformin 500 mg) for 15 days, and using the same CGM device were included. Those patients whose CGM devices came off before the target day were excluded. The patients were treated with metformin SR–vildagliptin FDC to achieve improved glycemic control. Patients with relatively lower baseline Hb_A1c_ levels (around 6.5%) were shifted to this FDC to overcome issues like hypoglycemia or GI side effects associated with their previous therapy. The treatment response was assessed by the percentage of time spent in the target glucose range, i.e., TIR at baseline and after 15 days of treatment with the metformin SR–vildagliptin FDC. The confidentiality of patients’ personal data was maintained. Treatment adherence and AEs, if any, were also recorded.

### 4.3. Results

The mean age of the population was 51 years, and all 10 patients had comorbidities, the most common being hypertension. Out of the 10 patients, 4 (40%) were treatment naïve, while 6 (60%) were treated with other antidiabetic medications, including OADs or insulin therapy. Other antidiabetic medications at baseline included insulin therapy, sulfonylurea monotherapy, and sulfonylurea plus metformin or alpha-glucosidase inhibitor plus metformin combination therapies. The HbA1c levels at baseline varied from 6.5% to 12% (mean HbA1c 8.7%). After treatment, the percentage of TIR improved from baseline in 7 of 10 patients (70%, mean increase in TIR: 18.9%), while it was nearly unchanged for 1 patient (10%) and decreased from baseline in 2 patents (20%, mean decrease in TIR: 11.0%). Treatment adherence was 100%. No GI symptoms or other AEs were reported. The patient details recorded at baseline and the treatment response are presented in Table 1.

### 4.4. Discussion 

Overall, treatment with metformin SR–vildagliptin FDC yielded good glycemic control in adult patients with T2DM having comorbidities, including patients who were uncontrolled with other OADs. The glycemic control improved in 70% of the participants. While 5% improvement in TIR is considered to be clinically meaningful [9], this FDC yielded about 19% improvement in TIR. Notably, all of these patients had one or more comorbid conditions, and 60% of the patients had received treatment with other antidiabetic medications. Further, the treatment was safe and tolerable, with no report of GI AEs, which possibly accounted for 100% treatment adherence. These findings indicated that the use of metformin SR–vildagliptin FDC in T2DM patients demonstrated striking improvement in GI tolerability and treatment adherence, unlike metformin IR–vildagliptin FDC, as reported by Mohan et al. [4]. In summary, these real-world data suggest that treatment with metformin SR–vildagliptin FDC for 15 days yields considerable improvement in TIR among patients with T2DM and is safe and well tolerated with a high treatment adherence rate.

#### Opinion on the Timing of Administration of Metformin SR–Vildagliptin Combination

Upon food intake, GLP-1 secreted from the gut enhances insulin secretion in a glucose-dependent manner, thus decreasing serum glucagon levels and improving hyperglycemia [27,28,29]. Inhibition of DPP-4 by vildagliptin prevents endogenous degradation of the incretin hormone GLP-1, thereby increasing plasma levels of its active forms [30,31,32]. The positive outcome of prior-meal ingestion of vildagliptin is evident in several clinical studies [30,33,34]. In a clinical study, prior-meal ingestion of vildagliptin resulted in >90% suppression of DPP-4 activity, which was associated with increased levels of both GLP-1 and GIP at the beginning of the meal, and the effect was maintained for over 14 h. Therefore, prior-meal administration of vildagliptin is associated with sustained inhibition of plasma DPP-4 activity and increased meal/post-meal levels of GLP-1 and GIP along with significantly reduced endogenous glucose production [30]. Therefore, the metformin SR–vildagliptin FDC may also be prescribed before meals. The key summary points on the use of metformin SR–vildagliptin FDC in T2DM management are presented in Box 1.

Box 1 **Metformin SR–vildagliptin FDC in T2DM management: Key summary points.**

- Use of metformin SR instead of IR formulation in the FDC may help promote adherence to OAD therapy, resulting in improved clinical outcomes and GI tolerability.
- Early combination therapy of metformin SR and vildagliptin in patients with T2DM is associated with significant and clinically relevant HbA1c reduction, good tolerability, good patient compliance and treatment satisfaction.
- The FDC of vildagliptin and metformin SR is effective with good GI tolerability.
- Current evidence indicates that administration of vildagliptin before meals may ensure optimal effect. Thus, the metformin SR–vildagliptin FDC may be prescribed before meals.

### 4.5. Limitations

The authors acknowledge a few limitations of the study, including the low number of patients (only 10 patients included), involvement of only a single study center, and short study duration. Large-scale, real-world studies with long-term follow-up involving multiple centers across the country would be imminent to generalize these findings for the Indian population with T2DM.

## 5. Conclusions 

Early intervention with metformin SR–vildagliptin FDC in patients with T2DM ensures therapy satisfaction/compliance in terms of superior efficacy along with safety and tolerability. Though metformin SR is associated with better GI tolerability owing to slow release, the potential recommendation of FDC (metformin SR + vildagliptin) administration before meals needs to be evaluated. Based on the VERIFY and INITIAL trials, future studies could be planned to explore different aspects, such as the impact of the early combination on disease progression and β-cell function over time.

## Figures and Tables

**Table 1:** Details of the case reports and real-world effectiveness of metformin SR–vildagliptin FDC treatment in patients with T2DM.

Patient No.	Baseline Data				Concomitant Antidiabetic Medication	% of TIR	
	Age and Gender	Comorbidity	Duration of Diabetes	Hb_A1c_		At Baseline	After Treatment
1	49 years, male	Hypertension	13 years and 8 months	7.70%	Glimepiride	70%	78%
2	82 years, female	Hypertension	NA	12%	Insulin Humalog Mix 25,	22%	69%
					SU (gliclazide) + metformin combination therapy		
3	55 years, female	Hypertension, dyslipidemia	4 years and 11 months	6.50%	Metformin monotherapy	93%	82%
4	64 years, male	Hyperthyroidism	25 years and 7 months	6.80%	Combination of alpha-glucosidase inhibitor (voglibose) and metformin	42%	67%
5	47 years, male	Hypertension	4 years	11.30%	Nil	45%	34%
6	40 years, female	Hypertension, dyslipidemia	5 years	7.30%	Nil	94%	98%
7	20 years, female	Hyperthyroidism	1 year and 7 months	10.20%	Insulin (mixtard 30/70, a biphasic insulin);	61%	65%
					combination of SU (gliclazide) + metformin		
8	53 years, male	Hyperthyroidism	5 years	9.00%	SU (gliclazide) monotherapy	70%	80%
9	52 years, female	Hyperthyroidism, dyslipidemia	9 years and 11 months	8.30%	Nil	66%	100%
10	48 years, female	Dyslipidemia	1 year and 8 months	7.90%	Nil	88%	87%

Hb_A1c_: Glycated hemoglobin; NA: Not available; SU: Sulfonylurea; TIR: Time in range.

## Data Availability

Not applicable.
